# RhoA as a Key Regulator of Innate and Adaptive Immunity

**DOI:** 10.3390/cells8070733

**Published:** 2019-07-17

**Authors:** Matthias Bros, Katharina Haas, Lorna Moll, Stephan Grabbe

**Affiliations:** University Medical Center Mainz, Department of Dermatology, Langenbeckstraße 1, 55131 Mainz, Germany

**Keywords:** small GTPases, RhoA signaling, immune cells, autoimmune diseases, infection, inflammasome

## Abstract

RhoA is a ubiquitously expressed cytoplasmic protein that belongs to the family of small GTPases. RhoA acts as a molecular switch that is activated in response to binding of chemokines, cytokines, and growth factors, and via mDia and the ROCK signaling cascade regulates the activation of cytoskeletal proteins, and other factors. This review aims to summarize our current knowledge on the role of RhoA as a general key regulator of immune cell differentiation and function. The contribution of RhoA for the primary functions of innate immune cell types, namely neutrophils, macrophages, and conventional dendritic cells (DC) to (i) get activated by pathogen-derived and endogenous danger signals, (ii) migrate to sites of infection and inflammation, and (iii) internalize pathogens has been fairly established. In activated DC, which constitute the most potent antigen-presenting cells of the immune system, RhoA is also important for the presentation of pathogen-derived antigen and the formation of an immunological synapse between DC and antigen-specific T cells as a prerequisite to induce adaptive T cell responses. In T cells and B cells as the effector cells of the adaptive immune system Rho signaling is pivotal for activation and migration. More recently, mutations of Rho and Rho-modulating factors have been identified to predispose for autoimmune diseases and as causative for hematopoietic malignancies.

## 1. Introduction

The rat sarcoma (Ras) superfamily of small guanosine triphosphate (GTP) hydrolase enzymes (GTPases) comprises over 100 members, and Rho family proteins constitute one of the five major branches of this superfamily with 25 proteins encoded by 22 genes in humans and mice [1]. Most Rho GTPases are subgrouped to various Rho families based on their degree of homology. In general, Rho GTPases constitute rather ubiquitously expressed cytoplasmic molecular switches which control the activity of an array of downstream effectors which, in turn, orchestrate a variety of cytoskeletal functions [2]. Thus far, the Rho subfamilies Rho (Ras homolog gene family) [3], Rac (Ras-related C3 botulinum toxin substrate) [4], and Cdc42 (cell division control protein 42 homolog) [5] which, besides Rif (Rho in filopodia) [6], represent the group of classical Rho GTPases have been studied most intensely. All of these have been shown to contribute to immune cell functions, and functional defects of single GTPases, as well as GTPase regulating factors, have been reported to result in serious immune dysfunction [4,7].

Activated immune cells require dynamic reorganization of their actin cytoskeleton to exert their functions, like phagocytosis of pathogens, migration, and cell-cell interaction [8]. Hence, all of these processes are regulated, in large part, by small GTPases [9]. This review aims to highlight the functional role of the prototypic GTPase RhoA and of components of the RhoA signaling cascade for innate immune cell types of the myeloid hematopoietic lineage that act as “sentinel” cells to detect and directly eliminate pathogens, comprising neutrophils, monocytes/macrophages, and conventional dendritic cells (DC). In addition, the involvement of RhoA proteins in the functions of adaptive immune cells, namely pathogen-specific T cells and B cells, that are recruited to convey an adaptive pathogen-specific immune response are highlighted.

## 2. RhoA Signaling

**Figure 1:**
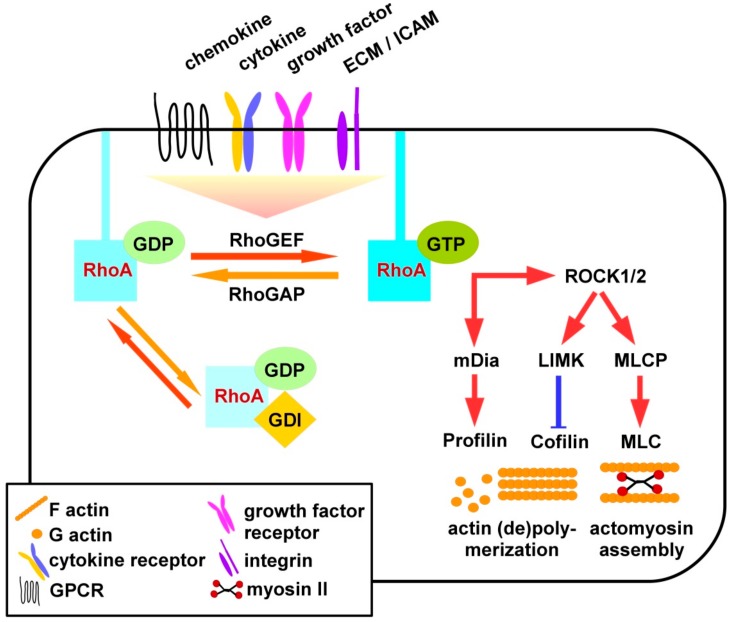
Scheme of RhoA signaling. Binding of exogenous ligands via different types of receptors as well as intracellular events trigger activation of RhoA GEF which, in turn, engage membrane-bound RhoA and mediate the exchange of GDP by GTP resulting in RhoA activation. GAP elevate the GTPase activity of RhoA, thereby promoting its inactivation. GDI translocate RhoA from the membrane and keep it in an inactive state. Active RhoA via protein kinases regulates cytoskeletal rearrangements. Active RhoA via ROCK/LIMK negatively regulates cofilin, which is required for F-actin turnover. Additionally, ROCK via inhibition of MLCP confers activation of MLC promoting actomyosin assembly. Active RhoA also promotes mDia activity which, in turn, activates profilin that is also involved in actin remodeling.

As depicted in Figure 1, RhoA is active when binding GTP, and inactive upon its hydrolysis to GDP [10]. RhoS signaling is triggered by binding of extracellular ligands as chemokines to G-protein linked receptors (GPCR), cytokines, and growth factors to their according receptors, extracellular matrix (ECM) factors and intercellular adhesion molecules (ICAM) to integrins. In addition to receptor engagement intracellular contacts may also differentially activate three classes of Rho GTPase regulating factors [11]. GEF (guanine nucleotide exchange factors) promote the exchange of GDP by GTP and, thereby, RhoA activity [12], and are counteracted by GAP (GTPase-activating proteins) [13] and GDI (guanine nucleotide-dissociation inhibitors) [14]. 

Most GEF belong to the diffuse B-cell lymphoma (Dbl) or to the dedicator of cytokinesis (Dock) protein family [15]. GEF of the Dbl family catalyzes nucleotide exchange via the Dbl homology domain (DH), and interact with other proteins and phospholipids via a pleckstrin homology domain (PH). Dock family members are characterized by Dock homology region (DHR), exerting catalytic activity via a DHR2 and engage proteins/phospholipids via a DHR1 domain. GEF activity is regulated predominantly by phosphorylation resulting in their inactivation or activation. Phosphorylation events can directly modulate GEF activity or affect the recruitment of additional proteins that regulate the activity of GEF [16]. An example for a GEF is Vav (named according to the sixth letter of the Hebrew alphabet) that is directly activated upon phosphorylation [12]. Vav appears in three isoforms, namely Vav1, Vav2, and Vav3 [17]. Vav1 is mainly expressed in hematopoietic cells while Vav2 and Vav3 show a broader expression pattern. Vav is involved in the regulation of cell migration, proliferation of cancer cells, phagocytosis, production of superoxide in neutrophils, and T-cell activation [16]. Importantly, Vav2 acts downstream of the transforming growth factor β (TGF-β) receptor and the epidermal growth factor receptor [16]. Myosin IXb (Myo9B) is an example for a RhoA-specific GAP [18]. Myo9B is a single-headed molecular motor containing a Rho-specific GAP domain, a Zn^2+^ binding site, and four binding sites for myosin light chains, and is highly expressed in leukocytes [19,20,21,22].

RhoA is associated to the membrane where it interacts with effectors [23]. In addition to GEF and GAP, RhoA can be regulated by GDI. These confer dissociation of RhoA from the membrane and maintain it in the cytosol. GDI also inhibit nucleotide exchange and GTP hydrolysis of RhoA [24]. The group of mammalian GDI consists of three members: RhoGDIα, RhoGDIβ, and RhoGDIγ. RhoGDIβ is expressed in hematopoietic tissues, especially in B- and T-lymphocytes [24]. 

In case of activation, RhoA interacts with downstream effectors such as Rho-associated protein kinases (ROCK), ROCK1 and ROCK2. These are serine-threonine kinases encoded by two different genes [25]. The N-terminus contains the catalytic kinase domain next to a coiled-coil region including the Rho-binding domain (RBD) and a PH domain [25]. ROCK1 and -2 also exhibit auto-inhibitory activity through binding of the C-terminus to the N-terminus. Active RhoA binds to the RBD of ROCK and activates it by disrupting the auto-inhibitory activity of the N- and C-terminal binding. The N-terminal kinase domain of ROCK is released and activated [25]. ROCK 1 and -2 are both expressed in most tissues but only ROCK1 appears to be expressed in the thymus and by leukocytes [26]. Activated ROCK inhibits myosin II regulatory light chain (MLC) phosphatase (MLCP) activity in a phosphorylation-dependent manner. In turn this results in elevated levels of phosphorylated and thereby active MLC mediating actomyosin assembly to induce the formation of stress fibers and focal adhesions [26]. Active RhoA also triggers phosphorylation-dependent activation of LIM domain kinase (LIMK) which, in turn, phosphorylates and thereby inhibits activity of cofilin [27]. Non-phosphorylated cofilin at low concentration promotes depolymerization of F-actin, whereas F-actin assembly is mediated at high levels of cofilin activity [28].

Furthermore, the RhoA/ROCK pathway controls cell migration, proliferation, and gene expression either through mediating nuclear localization of transcription factors or through direct regulation of the activity of transcription activators by phosphorylation [25]. In addition to the activation of ROCK downstream cytoskeletal effectors, active RhoA also binds mammalian diaphanous-related formin 1 (mDia1), thereby altering its auto-inhibitory state [29]. This allows binding of profiling which mediates actin polymerization.

## 3. Role of RhoA in Immune Cell Differentiation and Activity

Innate immune cells of the myeloid lineage like neutrophils, monocytes, and macrophages (MAC), and conventional dendritic cells (DC) share an overlapping repertoire of functions which they exert at varying efficacy. For example, neutrophils, and MAC are considered more efficient to phagocytose pathogens than DC [30]. Furthermore, at activated state DC constitute the most potent type of antigen presenting cells (APC), solely capable to activate naive antigen-specific CD4^+^ and CD8^+^ T cells, whereas MAC may only be able to re-stimulate primed and memory T cells [31], and neutrophils, due to a lack of MHCII, solely stimulate CD8^+^ T cells [32]. However, these paradigms have been challenged by the observation of inflammation-induced plasticity of myeloid cell types: monocytes may differentiate to so-called inflammatory DC [33], and polymononuclear neutrophils (PMN) may acquire a DC-like immunophenotype and APC activity [34]. Therefore, RhoA-specific functions that have been elucidated in one myeloid cell type may be largely applicable to the others as well.

### 3.1. Myeloid Innate Immune Cells

#### 3.1.1. PMN

PMN are innate immune cells of the myeloid lineage that are rapidly recruited to sites of invading pathogens and local inflammation [35]. There, immigrated PMN kill pathogens by various mechanisms including phagocytosis, and release proinflammatory mediators that shape and enhance the immunological defense [36]. PMN are attracted by various chemoattracting factors that are released by bacteria and chemokines derived from activated non-immune and immune cells [37].

##### Migration

**Figure 2:**
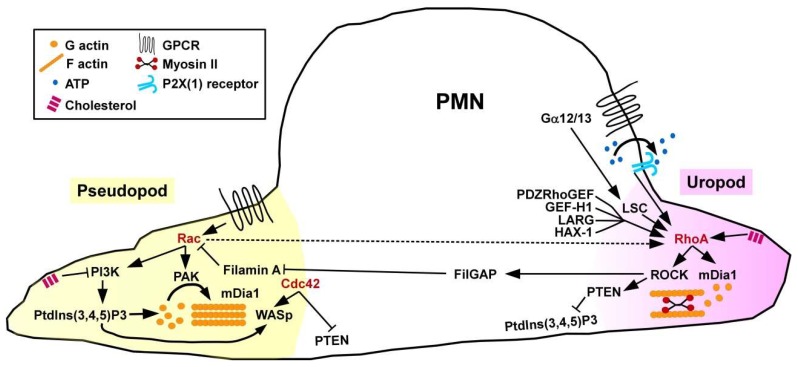
Spatially regulated activity of RhoA and other small GTPases is necessary for coordinated PMN migration. Chemoattractants induce Rac/Cdc42 activation at the migration front (pseudopod), and RhoA activty at the cells´ rear end (uropod). Spatial regulation of small GTPase activity is mediated by localized activity of GEF (e.g., LSC, GEF-H1), and GTPase-induced activation/inhibition of GAP (e.g., FilGAP), other adaptor factors, like PTEN, and PI3K signaling.

Sensing of chemoattractants by PMN results in cell polarization, namely the formation of a protruding pseudopod controlled by Rac GTPase that stimulates F-actin formation involving p21-activated kinase (PAK) as a downstream cytoskeletal effector protein [38], and of a retracting uropod regulated by active RhoA (Figure 2). Spatial activity of RhoA at the cell rear requires activity of the adaptor protein HS1-associated protein X-1 (HAX-1) [39] and of the RhoA GEF PDZRhoGEF [40] that governs actin-myosin II contractility. Unexpectedly, PMN derived from mice engineered to lack the F-actin cross-linking protein filamin-A were characterized by attenuated RhoA activity and uropod retraction in response to stimulation with the chemoattracting formylated tripeptide formyl-methionyl-leucyl-phenylalanin [41]. In line, in a peritonitis model, filamin A-deficient PMN showed strongly reduced infiltration into the peritoneum. Bacteria generate formylated peptides that are recognized by GPCR also expressed by PMN [42]). The G proteins Gα12/Gα13 proteins are activated by ligand-triggered GPCR and serve to translocate RhoGEF from the cytoplasm to the plasma membrane, and promote their GEF activity [43]. Both Gα receptors are coupled to the RhoA GEF LSC. PMN derived from LSC-deficient mice were characterized by disturbed formation of a dominant pseudopod [44]. However, in vivo PMN exerted unaltered migratory activity towards sites of inflammation suggesting compensatory mechanisms. In addition to chemoattractants, PMN also show migratory behavior in response to shear stress. In this regard, activation of RhoA-specific GEF-H1 has been implicated [45].

PMN migration requires mutual inhibition of Rac and RhoA which is mediated by several mechanisms. For example, RhoA via ROCK activation mediates phosphorylation of the Rac1-specific GAP FilGAP which binds filamin A localized at the cell front inhibiting Rac [46]. However, PMN obtained from Rac1 and Rac2 double-deficient mice were characterized by attenuated activity of RhoA at the uropod adding a level of complexity in counter-regulation [47]. Further, active Cdc42 which is also accumulated at the cellular front has been attributed to regulate the direction of PMN migration. Cdc42 and RhoA regulate spatiotemporal activity of phosphatase and tensin homolog (PTEN) which, in turn, regulates phosphatidylinositol 3-kinase (PI3K) activity, and, hereby, the direction of cell migration [48]. ROCK activity triggered by RhoA at the uropod complexes with PTEN and directly phosphorylates this protein. PTEN via dephosphorylation of the phospholipid PtdIns(3,4,5)P3 limits PI3K signaling at the posterior end of the cell. Bodin and Welch demonstrated that sequestration of the membrane lipid cholesterol supported uropod formation by inhibiting PI3K activity at the cellular front, and promoting RhoA signaling at the uropod at the same time [49]. As a positive (autocrine) feedback mechanism, ATP as released by chemo-attracted PMN was found to bind ATP-gated P2X(1) channels, which resulted in elevated RhoA activity [50]. In agreement, mouse PMN lacking P2X(1) receptors were characterized by attenuated uropod retraction and lower motility.

Concerning the role of GTPase downstream cytoskeletal effectors for migration, mDia1 regulates actin nucleation in response to chemoattractants [51]. mDia1^−/−^ PMN displayed attenuated activity of RhoA signaling components, including the Leukemia-associated Rho guanine-nucleotide exchange factor (LARG) [52]. Of note, mDia1 was also found co-localized with Wiskott–Aldrich syndrome protein (WASp) at the pseudopod.

#### 3.1.2. Macrophages

##### Polarization

Under steady state conditions macrophages (MAC) contribute to tissue homeostasis [53] by, e.g., clearance of apoptotic cells [54]. Depending on the nature of environmental signals polarization of steady state MAC (M0) towards so-called classical M1 or alternatively activated M2 macrophages has been described [55]. Phagocytic uptake of pathogens and detection of soluble danger signals results in the acquisition of a proinflammatory phenotype and, accordingly, polarized MAC are often termed M1 [56]. M1-type MAC migrate to sites of infection and clear pathogens by phagocytosis and extracellular effector mechanisms like reactive oxygen and nitrogen species (ROS, RNS). Furthermore, M1 MAC release proinflammatory cytokines like TNF-α to spread the alarm, and activate pathogen-specific T cells. In contrast to proinflammatory cues as mediated by pathogens and endogenous danger signals that result in M1 polarization of MAC, protolerogenic signals cause differential polarization of M0 MAC towards M2 [57]. M2-type MAC acquire antiinflammatory properties by releasing, e.g., interleukin (IL)-10 [58] but also type-2 innate cytokines, like IL-4 and IL-13 [59], to support wound healing [60]. However, these factors also contribute to allergic reactions [58]. Moreover, tumor-induced M2 MAC favor tumor growth directly, e.g., via release, e.g., of vascular endothelial growth factor-2, and indirectly, e.g., by promoting the generation of myeloid derived suppressor cells that inhibit anti-tumor immune responses [61]. Liu and co-workers [62] assessed the phenotype of differentially polarized murine bone marrow-derived RhoA^−/−^ MAC. Of note, non-polarized M0, as well as M2-polarized MAC, but not M1 MAC, were characterized by a strongly elongated morphology. All MAC subpopulations displayed morphological alterations of the Golgi complex [63].

##### Migration

**Figure 3:**
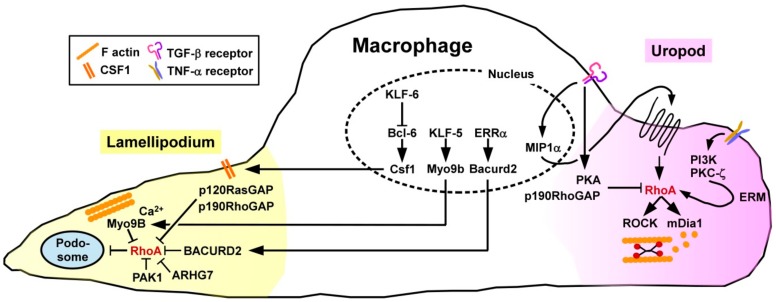
Migration of MAC requires spatially controlled activation of RhoA. At the migration front various GAP (e.g., Myo9B, p120RasGAP) and other factors (e.g., BACURD2) that inhibit RhoA activity. Endopeptidases concentrated in podosomes mediate dissociation of the ECM which is necessary for MAC migration. Podosome formation is inhibited by RhoA. At the rear end RhoA activity is required for uropod retraction. RhoA is activated, e.g., by TNF-α via PI3K/PKC-.ζ signaling resulting in activation of ERM which, in turn, translocate RhoA to the membrane. TGF-β first promotes RhoA activity via an autocrine MIP-1α stimulation loop, but at later time points inhibits RhoA via PKA signaling and p190RhoGAP.

To account for potential compensatory mechanisms between Rho GTPase subfamily members, Königs and co-workers [64] studied the overall importance of this GTPase subfamily using genetically engineered mice lacking those factors in a monocyte/macrophage-specific manner. In agreement with the general function of active RhoA to control retraction of the cells´ rear end in directed migration, Rho-deficient MAC showed a defect to retract their uropod resulting in the arising of elongated tails. Directionality was not affected, while speed was actually enhanced. Migration of MAC requires local degradation of the extracellular matrix (ECM) which is facilitated by various endopeptidases that are highly concentrated in podosomes [65]. Podosome formation depends on local inhibition of RhoA via the Rho/Rac-specific GEF ARHG7 and PAK1 [66] (Figure 3).

Pixley and co-workers demonstrated that the transcriptional silencer B cell lymphoma-6 (Bcl-6) serves to inhibit RhoA activation in MAC by controlling expression of the surface macrophage colony stimulating factor receptor (CSF)1 [67] known to mediate the translocation of p120RasGAP and p190RhoGAP to the cell membrane [68]. Hence, inhibition of Bcl-6 in murine bone marrow-derived MAC resulted in a higher level of active RhoA, a higher cortical F-actin density, more focal adhesion complexes, and lowered motility. Expression of Bcl-6 in MAC is negatively regulated by the Krüppel-like transcription factor 6 (KLF6) [69]. Accordingly, KLF6^−/−^ MAC were characterized by diminished migratory activity.

The prototypic proinflammatory cytokine tumor necrosis factor-α (TNF-α) which is produced by a large variety of activated immune cells [70] served to enhance the migratory activity of human monocytes as induced by chemokines [71]. Treatment with TNF-α activated several signaling pathways including PI3K and PKC-ζ resulting in phosporylation of the ezrin/radixin/moesin (ERM) cytoskeletal linker complex. Further, the ERM complex mediated translocation of cytosolic RhoA to the membrane. Treatment of MAC with transforming growth factor-beta 1 (TGF-β1), categorized as an anti-inflammatory cytokine generated by different tolerogenic immune cell types [72], was reported to differentially modulate the migratory activity of MAC in a time-dependent manner [73]. At shorter periods of treatment, TGF-β1 stimulated the migratory activity by inducing expression of the chemokine macrophage inflammatory protein-1α which in an autocrine manner facilitated RhoA activation. At later time points, TGF-β1 inhibited MAC migration by impairing RhoA activity via protein kinase A (PKA) induced phosphorylation and presumably by elevating p190RhoGAP activity.

More recently, the role of the RhoA GAP Myosin 9B (Myo9B) in migration of MAC has been highlighted. Myo9B is predominantly expressed in cells of the hematopoietic lineage, characterized by perimembrane localization, moving along F-actin until arrested at sites of elevated Ca2^+^ concentrations [74]. Due to conformational alterations, the Myo9B GAP domain is activated, thereby mediating RhoA inhibition in a fine-tunable spatiotemporal manner. In accordance, Myo9B^−/−^ MAC displayed higher levels of active RhoA, and consequently of phosphorylated and thereby inactive cofilin and MLC as the RhoA/ROCK cytoskeletal effectors [18]. Consequently, Myo9B^−/−^ MAC were characterized by defective polarization in response to chemoattractants, lacked the formation of lamellipodia, and were unable to migrate in a directed manner.

Ma and coworkers [75] identified the transcription factor KLF5 as a positive regulator of Myo9B gene expression. Myeloid-specific deletion of KLF5 in mice resulted in attenuated Myo9B expression, consequently an elevated overall RhoA activity in MAC, and concomitantly impaired their migratory activity. Overexpression of KLF5 promoted podosome formation in MAC, and increased their velocity. In the same study, human specimen of abdominal aortic aneurysms were demonstrated to contain elevated amounts of KLF5 and Myo9B considered as causative for enhanced MAC infiltration of the aortic wall.

While in vitro studies identified the RhoA GEF neuroepithelial cell transforming gene 1 (Net1) as important for MAC motility, in vivo deficiency of Net1 did not affect inflammation-induced MAC infiltration [76]. In addition to the role of RhoA-modulating GEFs/GAP, another signaling pathway comprises estrogen-related receptor alpha (ERRα) which upregulates expression of BTB/POZ domain-containing adapter for Cullin3-mediated RhoA degradation 2 (BACURD2) serving as an inhibitor of RhoA protein stability [77]. Hence, MAC derived from ERRα-deficient mice were characterized by elevated levels of active RhoA, impaired formation of lamellipodia and attenuated directional migration, while overall cell motility remained largely unaltered.

##### Phagocytosis

The role of small GTPases for phagocytic uptake of pathogens, apoptotic cells as well as immune complexes by myeloid immune cells has been studied primarily in macrophages. Phagocytosis of exogenous material is mediated either via Fc receptors (FcR) that bind antibody-opsonized material [78] or by the β2 integrin MAC-1 (CD11b/CD18) that recognizes complement-opsonized pathogens and, accordingly, was also termed CR3 (complement receptor 3) [79]. Moreover, MAC1 binds numerous serum components as well as ICAM-1 which is expressed by other immune cells to enable cell-cell contacts [80].

**Figure 4:**
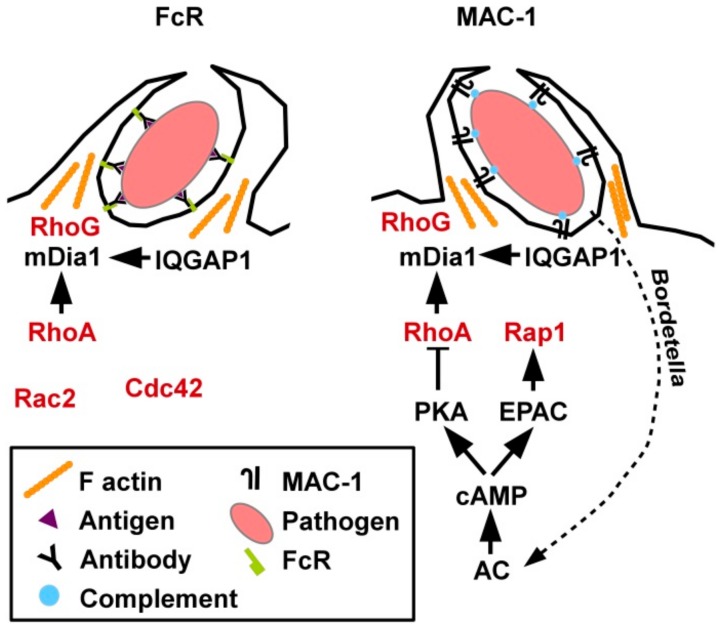
Phagocytic uptake of pathogens by MAC requires activity of RhoA and other small GTPases. Phagocytosis of opsonized pathogens is conferred by FcR recognizing the constant Fc part of antibodies which bind pathogen-specific surface antigens or by MAC-1 which binds activated complement deposited on the pathogen surface. In both cases partially overlapping sets of small GTPases are involved in phagocytic activity. As an evasion mechanism *Bordetella* bacteria generate toxins that trigger AC activity which inhibits RhoA.

Concerning the respective involvement of small GTPases in Fc receptor (FcR) versus MAC-1 mediated phagocytosis, individual depletion of GTPases by RNA interference revealed that FcR -dependent uptake required Rac2 and Cdc42, while CR3-mediated internalization involved RhoA [81], and Rap1 [82] (Figure 4). While conventional cyclic adenosine monophosphate (cAMP)-induced signaling inhibited RhoA activity [83], cAMP-induced exchange protein activated by cAMP (EPAC) activation triggered Ras-related protein 1 (Rap1) [82]. Hence, EPAC1 has also been termed Rap guanine nucleotide exchange factor 3 [84]. As observed by Kamanova and co-workers *Bordetella* bacteria which are phagocytosed via MAC-1 generated adenylate cyclase (AC) toxins [83]. Those induced sustained cAMP generation which resulted in RhoA inhibition, while the activity of other GTPases was not affected. The actin-polymerizing protein profilin was demonstrated to bind both active RhoA and Rap1. Moreover, active RhoG played a general role in phagocytosis, accumulating in the phagocytic cup irrespective of the uptake receptor (FcR or MAC-1).

In general, phagocytosis was demonstrated to require transient downregulation of active RhoA and a concomitant increase of active Rac1 as well as RhoG and Rab5 [85]. However, just prior to phagocytosis, enhanced RhoA activity was observed at the phagocytic cup [86]. At this site active RhoA is necessary to release mDia1 from auto-inhibition followed by binding of the cytoskeletal scaffold IQ domain-containing GTPase-activating protein 1 (IQGAP1) found highly enriched within the phagocytic cup [87].

#### 3.1.3. Dendritic Cells

##### Differentiation

DC comprise numerous subsets but can be can be broadly categorized as plasmacytoid (pDC) or conventional (cDC) DC that differ in their origin, lineage marker expression patterns and functional activities [88]. pDC mainly serve to sense viral infections resulting in release of high quantities of type I interferons to induce a broad anti-viral response. In contrast, cDC are considered to act predominantly as APC in case of infections as compared to pDC. Analysis of transgenic mice with a pan DC-specific deletion of RhoA revealed a reduction of the cDC compartment due to a higher rate of apoptosis [89]. Interestingly, as a compensatory mechanism the fraction of proliferating cDC was enhanced in case of RhoA deficiency, but this process was not directly regulated by RhoA. Further analysis identified PI3K-γ as strongly down-regulated in RhoA^−/−^ cDC. PI3K-γ signaling is known to inhibit apoptosis in leukocytes [90] which may explain the impaired long term viability of RhoA^−/−^ cDC.

##### Migration

**Figure 5:**
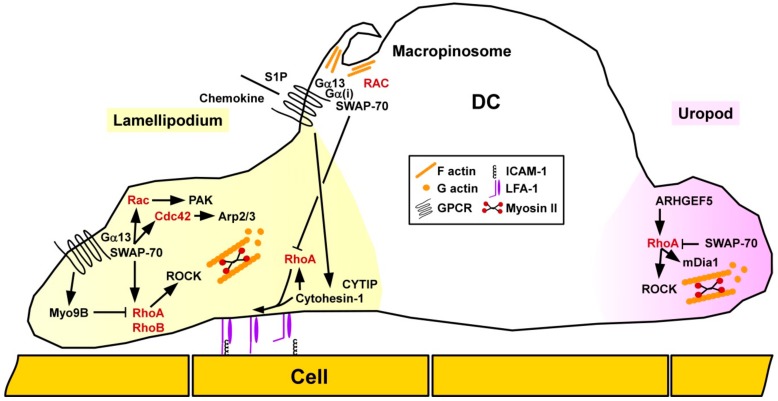
Dynamic regulation of spatial activity of small GTPases confers DC migration. DC migration requires coordinated activity of Rac/Cdc42 at the front and of RhoA at the rear end as regulated mainly by GEF (e.g., ARHGEF5) and GAP (e.g., Myo9B, SWAP-70). Active RhoA and CYTIP/Cytohesin-1 are necessary to mediate inside-out activation of LFA-1 to enable binding of ICAM and, thereby, cell-cell interaction.

Rho/ROCK signaling was shown to play an important role for migration of DC in vivo as pharmacological blockade of ROCK attenuated skin DC migration in a model of contact hypersensitivity (CHS) [91]. Concerning the spatial-temporal activity of small GTPases in DC migration, Vargas and co-workers reported that motile immature DC were characterized by accumulation of active Cdc42/Arp2/3 at the front and RhoA/mDia1 at the rear end [92] (Figure 5). As assessed by use of pharmacologic inhibitors and RNA interference, Cdc42/Arp2/3 limited the migratory capacity of unstimulated DC, but was required for antigen uptake. In response to stimulation, DC showed an increased motility which was associated with a decrease in Arp2/3. RhoA/mDia1 was necessary for chemokine-induced directed DC migration.

In DC RhoA was reported to modulate the activity of another β2 integrin family member, lymphocyte factor antigen-1 (LFA-1). Cytohesin-1 exerts both ADP-ribosylation factor GEF function and interacts with LFA-1 [93]. Quast and co-workers reported that Cytohesin-1 activated RhoA in DC, and both factors supported chemokine-mediated inside-out conformational activation of LFA-1 required for cell-cell contacts and migration [94]. Accordingly, depletion of Cytohesin-1 in DC by RNA interference affected DC migration. Similarly, human DC infected with the α-herpesvirus herpes simplex virus type 1 and the β-herpesvirus human cytomegalovirus were characterized by degradation of Cytohesin-1 interacting protein (CYTIP) which affected their migratory activity towards the chemokine CCL19 [95]. The authors suggested that herpesvirus-mediated CYTIP degradation constituted a viral immune evasion mechanism.

The switch-associated protein 70 (SWAP-70) has been recognized to act as a Rac GEF [96], but also as an F-actin bundling protein [97]. Ocaña-Morgern and co-workers demonstrated that SWAP-70 acts as an inhibitor of RhoA and indirectly also of RhoB activity in DC [98]. In DC, the phospholipid mediator S1P induces migration and endocytosis in DC [99]. SWAP-70^−/−^ DC displayed diminished upregulation of both functions in response to S1P treatment [100]. In that study, Gα(i) was found to engage SWAP-70 required for localization of Gα(13) in membrane rafts. Further, we have demonstrated that Rac-GTP/SWAP-70 are constituents of macropinosomes [101], which serve to internalize both soluble antigens and danger signals [102]. Concerning motility, SWAP-70^−/−^ DC displayed defective retraction of the uropod, and in general impaired redistribution of Rac and Cdc42 required for motility. Similar to the functional involvement of SWAP-70 in endocytic activity, Baranov and co-workers showed that this protein forms ring-shaped structures around the F-actin driven early phagocytic cup in DC, but in this case co-localized with Rac1 [103].

Unstimulated DC derived from mice deficient for the Rho-specific GEF ARHGEF5 were defective for chemokine-induced migration in vitro and displayed defective emigration from skin towards draining lymph nodes after application of the stimulatory dye fluorescein in vivo [104]. In agreement with impaired migratory activity, ARHGEF5-deficient mice were characterized by lower Th2 responses in a DC-dependent model of allergic airway inflammation.

We showed that deficiency of the RhoA-specific GAP Myo9b in murine DC resulted in a higher level of active RhoA, and elevate levels of phosphorylated and thereby inactive cofilin and MLC [105]. In agreement with dysregulated spatiotemporal regulation of RhoA activity, in a three-dimensional collagen gel Myo9B^−/−^, stimulated DC were unable to migrate along a CCL21 chemokine gradient. These findings of impaired directional migratory activity of Myo9B^−/−^ DC are in broad accordance with previous findings on Myo9B-deficient MAC [18]. In accordance, skin DC showed lower migration towards draining lymph nodes in response to fluorescein application [105].

##### Activation and Interaction with T Cells

In order to assess the role of small Rho GTPases for DC functions, Shurin and co-workers transduced murine DC with vaccinia virus that encoded either constitutively active or dominant negative mutated forms of RhoA, Rac1, and Cdc42 [106]. In these assays, RhoA had no effect on chemokine-induced migration, but promoted T cell stimulation as reflected by IL-2 contents of DC/CD4^+^ T cell co-cultures.

**Figure 6:**
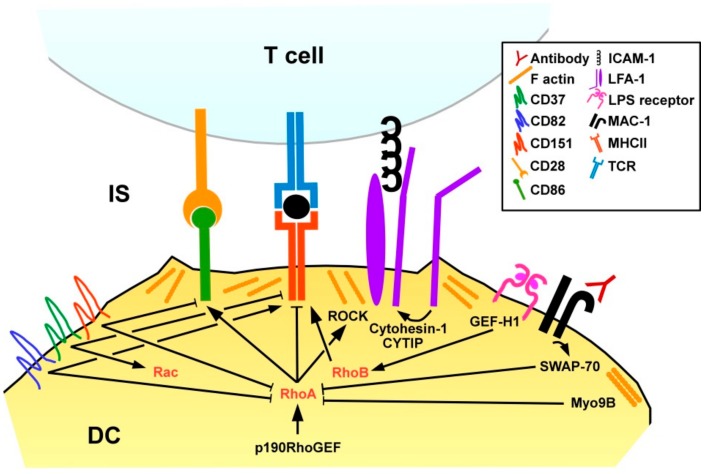
DC interact with T cells via the immunological synapse. Upregulated surface expression of the antigen receptor MHCII is inhibited by active RhoA, whereas RhoB may stimulate MHCII expression. RhoA is also involved in co-stimulatory expression. RhoA activity is regulated by GEF (e.g., p190RhoGEF) and GAP (e.g., SWAP-70, Myo9B), and by tetraspanins that also modulate MHCII/co-stimulator expression, presumably by other mechanisms. The duration of DC/T cell interaction is modulated by LFA-1 activity which is regulated by RhoA and CYTIP/Cytohesin-1.

DC present pathogen-derived peptides, termed antigens, via MHCI to CD8^+^ T cells, and via MHCII to CD4^+^ T cells [107]. Stimulation of DC with lipopolysaccharide was reported to promote translocation of major histocompatibility complex (MHC)II to the DC surface via activation of the RhoGEF GEFH1 which, in turn, stimulated RhoB activity, while RhoA was not found involved in that study [108] (Figure 6). In agreement with the RhoA/B inhibitory effect of SWAP-70 in DC, SWAP-70^−/−^ DC were characterized by strongly diminished MHCII surface expression, and poor CD4^+^ T cell stimulatory activity [98]. Inhibition of either RhoA or RhoB in SWAP-70^−/−^ DC was sufficient to restore MHCII surface translocation. In light of the previously mentioned study showing that LPS via GEF-H1/RhoB induced MHCII surface expression in DC [107] RhoB may exert differential activities on MHCII expression depending on the experimental context. SWAP^−/−^ DC displayed higher rates of cell-cell contact dependent spontaneous maturation as reflected by elevated expression of co-stimulatory molecules and MHCI-dependent CD8^+^ T cell stimulatory capacity [109]. Interestingly, antibody-induced MAC-1 signaling increased maturation of wild-type, but not SWAP-70^−/−^ DC, suggesting involvement of this β2 integrin. The importance of spatial-temporal regulation of RhoA for DC activity was also demonstrated by Seul and co-workers studying transgenic mice that overexpress the RhoA-specific GEF p190RhoGEF [110]. DC of these mice showed a perturbed activation pattern after stimulation, namely impaired migratory activity and upregulation of co-stimulators.

The interface between an APC and a T cell is termed immunological synapse (IS) and comprises the MHC/antigen complex that is recognized by the T cell receptor (TCR), co-stimulatory receptors like CD86 that engage CD28 as well as additional APC/T cell receptor pairs that mainly provide cell adhesion [111]. The IS of antigen-loaded Myo9B^−/−^ DC and co-incubated antigen-specific CD4^+^ T cells was characterized by lower F-actin contents on the DC side in case of Myo9b deficiency. Myo9B^−/−^ DC developed less cell-cell contacts with CD4^+^ T cells, whereas the duration of Myo9B^−/−^ DC/T cell contacts was enhanced as assessed in collagen gels. In accordance with altered DC/T cell contact parameters Myo9B^−/−^ DC induced lower T cell proliferation. In agreement with impaired migratory and T cell stimulatory activity of Myo9B-deficient DC, Myo9B knock-out mice showed lower CHS reactions in a skin allergy model.

In addition to GEF/GAP-mediated regulation of RhoA, tetraspanin proteins have also been reported to affect RhoA activity in DC. Tetraspanins are localized in the cell membrane and interact both with each other and various other proteins to regulate membrane protein organization and intracellular signaling [112]. Murine DC lacking CD37 showed elevated MHCII surface levels, whereas CD151^−/−^ DC expressed co-stimulatory molecules at higher extent [113]. In tumor cells CD151 was reported to limit RhoA activation [114]. Furthermore, CD82 was demonstrated as upregulated in the course of mouse DC activation and conferred RhoA inhibition [115]. However, in case of CD82 deficiency DC displayed diminished MHCII surface expression, and attenuated engagement of CD4^+^ T cells. In the same study, CD37 was reported as down-regulated upon DC stimulation, and to act as a Rac1 activator, conferring motility of unstimulated DC. (Interestingly, CD37 expression was required for migration of DC induced by interaction of CLEC-2 with podoplanin which is expressed by lymph node stromal cells [116].) In contrast to most RhoA-modulating GEF/GDP the expression pattern of tetraspanin proteins considerably differs between mice and humans, and between different DC subpopulations [117]. Therefore, additional studies are necessary to elucidate the functional role of tetraspanins in small GTPase functions in DC in a species- and population-dependent manner.

Hofer and co-workers reported on CYTIP induction in stimulated DC [118]. CYTIP accumulated in the IS and was found necessary to promote detachment from contacting T cells. Under conditions of low antigen presentation, DC with depleted CYTIP exerted diminished T cell stimulatory activity. We have shown that LFA-1 on DC inhibited the extent of DC-induced T cell activation, and that CYTIP served to keep LFA-1 on DC in an inactive state to allow T cell stimulation [119].

### 3.2. Adaptive Immune Cells

#### 3.2.1. T Cells

##### Differentiation

RhoA activity is important for thymocyte differentiation as observed in mice with T cell compartment specific deletion of RhoA [120]. In these mice, thymocytes at all developmental stages showed elevated levels of apoptosis due to increased ROS production. Overproduction of ROS was a consequence of enhanced metabolic mitochondrial activity. In accordance, treatment of transgenic mice with the ROS scavenger N-acetylcysteine [121] largely rescued thymocyte differentiation. Furthermore, the RhoA-GAP ARHGAP19 was identified as preferably expressed in T cells, mediating translocation of myosin II and of citron to the plasma membrane [122]. In addition, ARHGAP19 via RhoA/ROCK2 transmitted phosphorylation of vimentin required for the maintenance of cell shape. Overexpression of ARHGAP19 inhibited cytokinesis as necessary for cell division.

##### Migration

**Figure 7:**
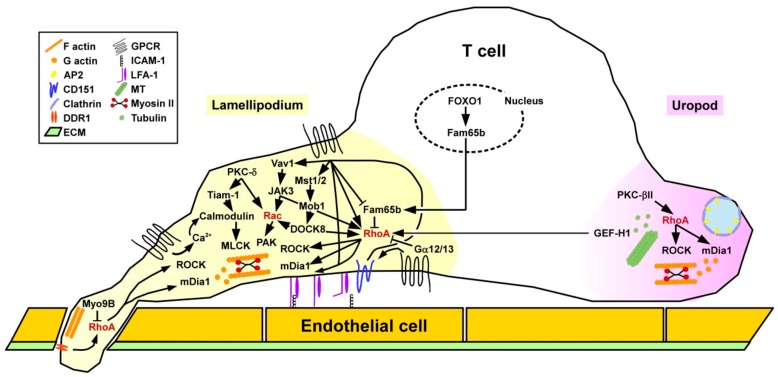
Migration of T cells is regulated by spatially regulated activity of RhoA and other small GTPases. Chemokine-induced T cell migration is conferred by dynamic regulation of the small GTPases RhoA/Rac at the front and of RhoA at the rear end. The activity of small GTPases is differentially regulated by GEF (e.g., GEF-H1), GAP (e.g., Myo9B), and other factors (e.g., JAK3, Fam65b, PKC). Microtubuli turnover and the presence of clathrin structures contribute to the migratory activity.

Binding of chemokines to T cells causes intracellular mobilization of the secondary messenger Ca2^+^ which triggers calmodulin, and downstream myosin light chain kinase (MLCK) resulting in rearrangements of the acto-myosin cytoskeleton to confer cell protrusion [123,124] (Figure 7). At the same time, RhoA and downstream ROCK activity are induced at the rear end of the cell to enable detachment and retraction. T cells require active RhoA at the leading edge within lamellipodia and filopodia, regulating protrusion via mDia1 and retraction via ROCK, which involves differential spatial activity of various RhoA GEFs [125]. In addition to regulation of F-actin at the lamellipodium, RhoA/ROCK signaling is also necessary to form microtubules (MT) at the uropod. The MT-associated RhoGEF GEF-H1 when released from the MT serves to activate RhoA in lamellipodia of migrating T cells [126].

Furthermore, as mentioned above RhoA is implicated in the conformation-dependent acquisition of a high affinity state of LFA-1 via inside-out signaling [127] as a prerequisite for binding to ICAM-1 expressed by endothelial cells and APC [128]. Similar to myeloid cell types, chemokine-mediated activation of RhoA in T cells increased the binding affinity of the β2 integrin LFA-1 to cellular receptors in order to mediate T cell adhesion to high endothelial venules via binding to vascular cell adhesion protein 1 [129] and contact with other immune cells like APC by engagement of ICAM-1 [127]. Within lymph nodes, besides chemokines, lysophosphaticidic acid (LPA) that binds to various GPCR triggered RhoA activity required for T cell migration [130]. LPA is generated from extracellular lysophosphatidylcholine by the ectoenzyme autotaxin that is expressed and deposited on the surface of stromal cells [131]. Migration of activated T cells through the (ECM) requires activtiy of the collagen I receptor DDR1 (discoidin domain receptor 1) triggering the RhoA/ROCK/ERK signaling pathway [132]. After infiltration of inflamed tissue, activated T cells were characterized by elevated expression of the RhoA downstream effector mDia and the cell cytoskeletal effector profilin accumulated at the leading edge [133]. mDia-induced actin polymerization contributed to block chemotaxis.

In addition to RhoA-mediated inside-out LFA-1 activation as, e.g., induced by CCL21, other chemokines like C-X-C (motif) ligand (CXCL)9 triggered LFA-1 adhesiveness bypassing RhoA [127]. Chemokines were shown to trigger Vav1 in T cells, which activated Janus kinase 3 (JAK3), acting on RhoA [134]. Furthermore, RhoA in concert with phospholipase D (PLD) mediated activation of Rap1A. Furthermore, JAK3 also facilitated Rac1 activation [135]. Accordingly, pharmacological inhibition of JAK3 inhibited the formation of lamellipodia and a uropod as well as the migratory activity of T cells. Furthermore, members of the mammalian sterile twenty (Mst) kinase family [136] represent positive regulators of T cell adhesion and migration as shown in transgenic mice with a double deficiency for Mst1/2 [137]. In these mice differentiated T cells accumulated in the thymus due to a defect in emigration. Mst1/2 was necessary to transmit CC-chemokine ligand (CCL)21-mediated phosphorylation of Mps one binder 1 (Mob1). In turn, Mob1 activated DOCK8 regulating RhoA and Rac1 activity. Interestingly, RhoA/ROCK signaling in migrating T lymphocytes was demonstrated to control the spatial localization of clathrin-rich structures at the uropod region where they co-localized with the endocytic receptor complex adaptor protein 2 (AP-2) [138]. In a myosin II-dependent manner these structures mediated endocytosis as shown for transferrin. Notably, inhibition of clathrin was sufficient to attenuate chemokine CXCL12-induced T cell migration.

Phorbol 12-myristate 13-acetate (PMA) is known to stimulate various members of the protein kinase C (PKC) family [9]. Under flow conditions, in PMA-stimulated T cells, on the one hand, active PKC-δ localized in lamellipodia and regulated T cell protrusions via the GEF T cell lymphoma invasion and metastasis 1 (Tiam1) [139], Calmodulin, and Rac1. On the other hand PKC-βII accumulated in the uropod and stimulated RhoA and thereby the stability of microtubuli (MT) [140]. Gα12 and Gα13 constitute negative regulators of RhoA, and thereby also affected RhoA-induced LFA activation [141]. Moalli and co-workers reported that CD8^+^ T cells deficient for the RhoGAP Myo9B while not affected in their *in vitro* motility showed attenuated migration into non-lymphoid tissues like the epidermis due to impaired crossing of the ECM between dermis and epidermis [142]. The migration defect of Myo9B^−/−^ CD8^+^ T cells caused impaired anti-viral responses in skin virus infection models.

Fam65b (family with sequence similarity 65 member b) was identified as an inhibitor of chemokine/RhoA-induced adhesion and migration [143]. Fam65b directly engaged RhoA and affected the GDP/GTP exchange. Chemokine binding induced phosphorylation of Fam65b and thereby attenuated its binding to RhoA [144]. In T cells, Fam65b expression was found to be controlled by the transcription factor FOXO1 (Forkhead box O 1) [143]. FOXO1 is well known to dampen T cell responses [145]. By a yet unknown mechanism co-culture of chronic lymphocytic leukemia cells of patients with autologous T cells attenuated RhoA and Rac1 activity, but elevated Cdc42 activation [146]. Similar to DC, tetraspanins have been attributed an important role for T cell functions. CD151 was reported to co-localize with LFA-1, and in response to CCL21 treatment contributed to tyrosine phosphorylation of Vav1, actin polymerization, and directed migration.

##### Activation

The stimulation of T cells by APC requires the formation of an IS [147]. High-resolution microscopy revealed that naïve CD4^+^ T cells due to attenuated cytoskeletal flexibility formed IS of smaller diameter than observed for T effector cells [148]. This observation correlates with the requirement of higher APC activity required for stimulation of naïve T cells as compared with (re)stimulation of effector and memory T cells [149]. Thauland and coworkers showed that naïve CD4^+^ T cells displayed higher Rho/ROCK activity and, hence, attenuated activity of cofilin required for F-actin turnover than effector T cells. In line, pharmacological inhibition of RhoA signaling in naïve T cells increased the IS size. Inhibition of cofilin activity and forced expression of active ROCK in effector T cells also decreased the IS size and attenuated T cell stimulation.

**Figure 8:**
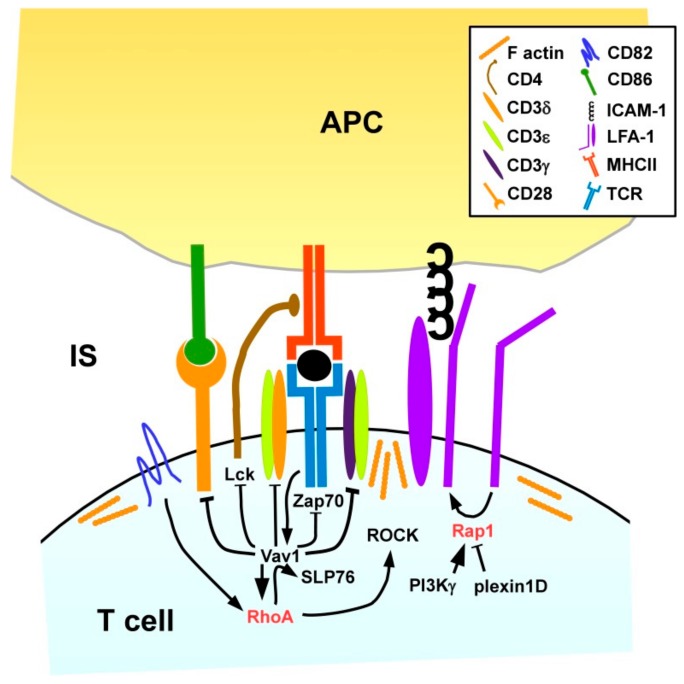
Activation of antigen-specific T cells by APC via receptor pairs localized within the IS results in RhoA activation. Recognition of the MHC/antigen complex by the TCR, concomitant co-stimulation via CD86/CD28 and ICAM/LFA-1 dependent adhesion results in activation of the Rho/Rac GEF Vav1. CD82 further promotes Vav1 activation/interaction with SLP76 in a RhoA-dependent manner. Active Vav1 activates small GTPases, and inhibits TCR components and CD28 to prevent excessive T cell stimulation.

We and others have shown that LFA-1 on T cells needs to contact ICAM-1/2 on the APC for proper IS formation (Figure 8), since LFA1-deficient CD4^+^ T cells displayed attenuated activation which was rescued by antibody-mediated TCR stimulation [150]. Rap1 has been identified as a positive regulator of LFA-1 activity [151], and as outlined above, cross-talk of Rap1 and RhoA has been reported [83,134,152]. In several studies factors that positively (PI3Kγ [153]) and negatively (plexin D1 [154] regulate Rap1, respectively, have been identified.

As outlined above, in DC the tetraspanin CD82 is required for MHCII surface expression and T cell engagement [115]. Likewise, CD82 was found to accumulate within the IS on the T cell side [155]. The Rho/Rac GEF Vav1 is known to be rapidly tyrosine phosphorylated and thereby activated after stimulation of the TCR when binding a MHC/antigen peptide complex at high affinity [17]. Delaguillaumie and co-workers demonstrated that CD82 in a RhoA-dependent manner promoted the tyrosine phosphorylation-induced activation and association of Vav1 and the Src homology 2 (SH2) domain-containing leukocyte protein of 76 kDa (SLP76) in response to T cell activation [155]. Active Vav1 in turn prevented phosphorylation of several components of the T cell receptor (TCR) signaling complex, including TCR-associated CD3 δ/ε/γ chains, lymphocyte cell-specific protein tyrosine kinase (Lck) associated with the TCR co-receptor CD4 and the ζ chain of TCR-associated protein kinase 70 (ZAP70) as well as of CD28 [156] which interacts with co-stimulatory receptors on the APC surface exerting co-stimulation as necessary for full T cell activation [149]. Therefore, RhoA/Vav1 are critically implicated in the intricate negative feedback loop of T cell stimulation [157].

In addition to direct effects of small GTPases on gene expression via cytoskeleton-associated transcription factors like Serum response factor (SRF) [158], RhoA was also demonstrated to inhibit the transactivation properties of the transcription factor nuclear factor of activated T-cells (NFAT) at the IL-2 promotor site, and to impair acetylation of histone A3 at this promoter, thereby interfering with IL-2 expression after T cell stimulation [159].

#### 3.2.2. B Cells

##### Differentiation

Genetic deletion of RhoA in hematopoietic stem cells of mice was shown to reduce the frequencies of B cell progenitors (proB, preB) and of immature B cells in the bone marrow [160]. In the same study, inhibition of RhoA expression specifically in CD19^+^ B cells affected the numbers of splenic B cell populations. The functional role of RhoA for B cell differentiation and development was confirmed by Ishizaki and co-workers demonstrating that dual deletion of Rho GDIα and β in mice resulted in lower levels of splenic marginal zone B cells [161].

##### Activation

**Figure 9:**
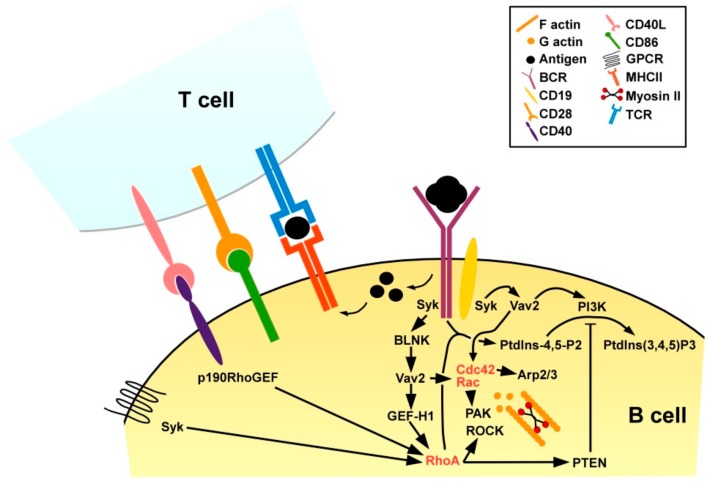
BCR activation and concomitant T cell help activate RhoA and other small GTPases in B cells. Binding of a protein antigen by an antigen-specific BCR supported by the co-receptor CD19 triggers Syk which, in turn, activates Vav2 mediating, e.g., via the GEF GEF-H1 activation of RhoA, and of other small GTPases, as well as PI3K activation. RhoA via PTEN negatively regulates PI3K. Antigen-specific CD4^+^ T cells that bind antigen presenting B cells and receive co-stimulation via CD86/CD28 transiently upregulate CD40L. Binding of CD40 by CD40L activates RhoA via p190RhoGEF. In addition, chemokines may stimulate RhoA.

RhoA is also an important regulator of B cell activation. Engagement of the B cell receptor (BCR) by a protein antigen results in redistribution of phosphatidylinositol-4-phosphate 5-kinases to the plasma membrane for local synthesis of phosphatidylinositol-4,5-bisphosphate (PtdIns-4,5-P2) [89] (Figure 9). In turn, PtdIns-4,5-P2 is required for the production of secondary messengers to transmit downstream signaling [162]. RhoA-deficient B cells failed to generate PtdIns-4,5-P2 in response to BCR engagement, and consequently displayed no calcium influx and proliferative activity [163]. Triggering of the BCR by engagement of a protein antigen binding at high affinity activates BCR-associated Syk (spleen tyrosine kinase) which via the B cell linker (BLNK) protein in turn activates Vav2 stimulating on one hand Rac and Cdc42, and on the other hand via GEF-H1 RhoA [164]. The BCR co-receptor CD19 in response to BCR stimulation in a Syk-dependent manner was found trigger Vav2 as well, thereby both activation of Rac and Cdc42 as well as of PI3K required for PtdIns-4,5-P2 processing [165]. Of note, RhoA-mediated PTEN activation [166] may limit PI3K activity [167] and thereby the extent of B cell activation. In addition to BCR engagement, B cell activation requires concomitant CD4^+^ T cell help [168]. For this, protein antigen internalized via the BCR is processed, and derived peptide antigens are presented via MHCII. Antigen-specific CD4^+^ T cells that recognize the presented antigen and receive co-stimulation, e.g., via CD86 transiently upregulate CD40L [169]. CD40L engages CD40 which is expressed by APC including B cells. CD40 receptor engagement was reported to induce expression of RhoA-specific p190RhoGEF [170] and is essential for the acquisition of a B plasma cell immune-phenotype [171]. RhoA activation in B cells has also been observed in response to triggering of chemokine receptors [172] downstream of the protein tyrosine kinase Syk [173].

## 4. Pathophysiological Role of RhoA Signaling in the Course of Immune Reactions

In consideration of the fact that immune reactions are based on a sophisticated interaction between hematopoietic and non-hematopoietic cells, a tight regulation of this interaction is crucial to ensure an effective elimination of pathogen and to suppress a misguided and exuberant immune response. Within the last years an increasing number of studies reported that RhoA and its downstream effector molecules have an essential function during infection processes [174,175,176,177,178] and in the onset and progression of various autoimmune diseases [179,180,181,182,183,184,185], such as multiple sclerosis [186,187,188,189,190].

### 4.1. The State of RhoA Activity Determines both Cellular Uptake and Elimination of Pathogens

An infection is caused by the successful entry of a pathogen into an organism and its propagation in the host [191]. For this purpose, microorganisms have developed a variety of sophisticated mechanisms during evolution aiming to interfere with immune responses to establish an infection [192]. For example, a favorite entry route relies on the rearrangement of the actin cytoskeleton by modifying the family of Rho GTPases as RhoA [175]. The inactivation of RhoA GTPase has multiple consequences on host cells, e.g., inhibition of cell migration or cell death, because RhoA is a master regulator of cell migration, cell cycle, and numerous immunological processes [193]. Recently, Alto and co-workers demonstrated [174], that the pathogens *Salmonella, Shigella* and enteropathogenic *E. coli* produce an effector protein which diminished the host cell function by imitating the Ras GTPases signaling pathway. This study clearly demonstrated, that the effector proteins IpgB1 and IpgB2 (*Shigella*), as well as Map (*E. coli*) functionally imitate the activated forms of distinct Rho family GTPases. IpgB2 mimics active RhoA and initiates the formation of stress fibers, while IpgB1 and Map act similar to the active forms of Rac1 and Cdc42. Furthermore, IpgB1 induces dorsal lamellipodia, and Map initializes the formation of cell surface filopodia [174]. All of these factors act to promote uptake of the pathogen by the host cell [178].

However, somatic cells possess a cell-autonomous innate immune signaling network as a first line of defense against invading pathogens [194]. The host cell expresses various sensor proteins, which identify bacteria or bacteria-specific products. As a consequence, transcriptional and post-translational responses are initiated [195]. Pyrin is an intracellular pattern recognition receptor that monitors the cytosol and detects disruption in cytoplasmic homeostasis triggered by invading bacteria. Activated pyrin formed a multiprotein complex, called inflammasome [175], which is an innate protein immune complex [193]. Under physiological conditions pyrin is inactive, because i) RhoA activates the serine/threonine-protein kinase N (PKN)1/2, which keeps pyrin in inactive state and ii) the protein complex 14-3-3ε is bound to pyrin. Interestingly, several bacteria-derived products, including TcdA and TcdB from *C. difficile*, VopS from *Vibrio parahaemolyticus*, or YopE and YopT from *Yersinia spec.*, inactivate RhoA [175]. As a consequence, PKN1/2 is dephosphorylated, which results in dissociation of 14-3-3ε from pyrin whereby the autoinhibition shall be suspended and the pyrin inflammasome can be formed [177]. In this context it must be pointed out that pyrin perceives the effects of bacterially induced RhoA modification and does not interact directly with RhoA. This was extensively studied by Shao and co-workers [176]. They showed that the major virulence factor TcdB from *Clostridium difficile* modifies RhoA via glycosylation. This modification of RhoA is recognized by pyrin and initiates the assembly of the pyrin inflammasome. As a consequence, caspase 1 is activated. The authors concluded that all bacterial products demonstrated to affect RhoA activity via glycosylation, adenylation, ADP-ribosylation, and deamidation on different residues of RhoA. The assembly of the pyrin inflammasome leads to the activation of Caspase-1, which supported the inflammation. Active caspase-1 ensures the proteolytic maturation and secretion of pro-inflammatory IL-1β [196] and IL-18. Furthermore, the necrotic type of cell death, named pyroptosis, is activated [193]. Consequently, the bacteria initiate their own elimination [178].

It can be concluded that pathogens modulate the cytoskeleton to enter the host cell [175] and that the pathogen-mediated inhibition of RhoA has two contrary effects: first, fundamental cellular processes including cell cytoskeletal organization are turned off, which results for example in an impairment of leucocyte migration [177]. Second, the inhibition of RhoA initiates inflammasome activation, which may result in the elimination of the bacterium [178]. After all, in the course of evolution pathogens and host have adapted to each other. Thus, it seems that this partnership based on a steady interplay between pathogen (action) and host (reaction), which allow the replication of the pathogen but, simultaneously, the host immune system suppresses an excessive growth and so an established infection is prevented.

### 4.2. Key Factors of the RhoA Signaling Pathway Constitute Targets for the Treatment of Autoimmune Diseases

The main hallmark of autoimmune diseases is a disturbance in immune tolerance, i.e., the immune system is no longer able to distinguish between self and non-self antigens. As a result autoreactive T cells and B cells are activated, which attack the organism [197]. In recent years the importance of RhoA signaling in autoreactive immune cells for the onset and progression of autoimmune diseases has come into the focus of research.

Multiple sclerosis (MS) is a complex chronic autoimmune disorder of the central nervous system [186,187,188,198], which is characterized by infiltrating autoreactive lymphocytes and mononuclear cells, which cause inflammation and demyelination [186,198] and, consequently, neurodegeneration of gray and white brain matter [188]. It has been extensively documented that T cells play a key role in the pathogenesis of MS [188,199,200,201]. In the periphery, T cells constantly circulate through lymphatic tissue, blood vessels [202,203] and the extravascular area to monitor the periphery [204] and play a critical role in the onset of autoimmune diseases [205]. In case of infection antigen-specific naïve T helper cells (Th) are activated by APC like DC [206,207,208]. As a result of T cell activation the Th0 cell [209,210,211] differentiates into one of the four major subsets (Th1, Th2, Th17, regulatory T cells [Treg]) [205,212]. Th1, Th2, and Th17 are important for the induction of adaptive immune response against pathogens, whereas Treg are considered to play an essential role in the control of autoreactive T cells and prevent excessive immune reactions that may harm the host [205]. However, a growing body of evidence suggests that Th17 play an essential key role in the pathogenesis of autoimmune disease [189,207,208,213,214], e.g., in experimental autoimmune encephalomyelitis (EAE) employed as a rodent model of MS [207]. Infiltration of the brain by autoreactive T cells depends on the rearrangement of the cytoskeleton [188]. A central protein in T cell migration is RhoA [188]. By now, several studies have pointed out that RhoA and downstream effector proteins of the RhoA/ROCK pathway are involved in the onset and progression of EAE [186,187,188,189].

Hasseldam and co-workers [188] aimed to characterize the function of RhoA in autoreactive T cells (Fig. 2). Studies on mice with a T cell-specific knock-out of RhoA revealed that this small GTPase is important for the i) proliferation, ii) activation and iii) migratory capacity of T cells. Noteworthy, the deficiency of RhoA in T cells led to a delayed onset of EAE as well as reduced disease severity. Another study investigated the function of the RhoA GAP Myo9B [18] in EAE, which is highly expressed in CD4^+^ T cells [19]. In mice deficiency of Myo9B in T cells was associated with a delayed onset of EAE-specific symptoms [187]. The delayed initiation of EAE was associated with a lower number of Th1 and Th17 cells as well as decreased levels of the pro-inflammatory cytokines IL6 and IFN-γ. Interestingly, during the recovery phase the number of Th1 and Th17 cells was increased, and the concentration of the cytokines IL1α, IL6, TNFα, and IFNγ was strongly raised in the brain. These data showed that the constitutive lack of Myo9B has two reciprocal effects: First, in the initiation phase the absence of Myo9B suppresses an excessive immune response, which resulted in delayed manifestations of EAE. Second, in the recovery phase the loss of Myo9B seemed to be over-compensated resulting in stronger inflammation.

Several single nucleotide polymorphisms (SNP) are associated with a higher risk to develop autoimmune diseases like systemic lupus erythematosus (SLE), rheumatoid arthritis (RA), celiac disease (CD) [185], and type 1 diabetes [184]. T cell activation Rho GTPase-activating protein (TAGAP) contains several SNP which are associated with progression of various autoimmune disease such as psoriasis [179], RA [180], Crohn‘s disease [181], CD [182,183], and MS [190]. Recently it could be demonstrated that TAGAP facilitated EAE disease severity and weight loss of mice [189]. Several studies reported that in mouse vitamin D and 1,25-dihydroxyvitamin-D_3_ (calcitriol, 1,25 (OH)_2_D_3_) had a positive impact on the progression of EAE [215,216,217,218,219]. Singh and co-workers demonstrated a link between the inhibition of the RhoA/ROCK pathway in autoreactive CD4^+^ T cells and vitamin D3 in EAE [186]. In EAE, autoreactive Th1 and Th17 deplete vitamin D [217]. Impairment of RhoA/ROCK signaling by lovastatin (LOV) restored vitamin D levels [186]. Interestingly, the administration of LOV in combination with vitamin D resulted in i) a shift from Th1 to Th2 responses; ii) attenuated frequency of Th17; and iii) increased Treg induction. These observations suggest that the RhoA/ROCK pathway constitutes an interesting target for the correction of the imbalance between Th17 cells and Treg as an immunological hallmark of MS [214]. Hence the conclusion can be drawn, that RhoA/ROCK and the corresponding downstream effector molecules might be suitable targets to develop new therapeutic approaches for the treatment of autoimmune diseases, such as MS [186,187,188,189].

## 5. Mutations of RhoA Signaling Components in Immune Cells Cause Malignancies

In several recent publications the pathophysiological importance of mutations of the RhoA GTP-binding pocket region for T lymphoma has been elucidated. Mutated RhoA G17V is apparent in numerous CD4^+^ T cell lymphomas characterized by a follicular helper T cell (Tfh) like immune-phenotype [220]. In accordance, transgenic mice engineered to express RhoA G17V driven by a CD4 gene promoter displayed attenuated levels of naive and enhanced frequencies of Tfh-like cells [221]. These mice developed autoimmunity attributed to the hyper-responsiveness of CD4^+^ T cells towards polyclonal stimulation. Fujisawa and co-workers reported that RhoA G17V mediated stronger Vav1 tyrosine phosphorylation which resulted in excessive TCR signaling [222]. In addition, a fraction of T cell lymphoma samples that was negative for the RhoA G17V mutation displayed gain-of-function mutations of Vav1. Incubation of lymphoma cells with the tyrosine kinase inhibitor dasatinib, which is clinically used for leukemia treatment [223], limited Vav1 phosphorylation, and TCR stimulation [222]. Infection of CD4^+^ T cells by the human T lymphotropic virus type 1 frequently resulted in mutations of the RhoA GTP binding pocket region like RhoA C16R which elevated the GTP/GDP exchange, while others exerted opposite effects [224].

In B cells RhoA may exert tumor suppressive functions as loss-of-function mutations of the guanine nucleotide-binding protein (G)α13 that activates predominantly RhoA-specific GEFs [16] frequently occur in diffuse large B cell lymphoma [225] and Burkitt´s lymphoma [226]. Similarly, numerous B cell lymphoma cell lines were characterized by inactivating mutations of the Gα13 effector ARHGEF1 [227], and of RhoA itself [226]. A knock-out of Gα13 in mice caused resistance of germinal center B cells towards caspase-dependent apoptosis and enhanced somatic hypermutations in the immunoglobulin VH gene locus [228]. Consequently, Gα13-deficient mice developed B cell lymphoma [227]. Recently, Bouafia and co-workers demonstrated that an inherited loss-of-function mutation of RhoA-specific ARHGEF1 in human was the cause for elevated frequencies of transitional B cells, accompanied by a lack of splenic marginal zone B cells and memory B cells [229]. Consequently, patients suffered from impaired antibody production and, hence, recurrent infections. Transduction of patients’ B cells with wild type ARHGEF1, as well as pharmacological RhoA activation in vitro, rescued defects in actin cytoskeleton remodeling and PI3K signaling.

## 6. Concluding Remarks

By now the importance of RhoA signaling in both innate and adaptive immune cell types for virtually all aspects of their functional activity has been firmly established. Similar to other cell types, in immune cells RhoA was shown to (i) be controlled by GEF and GAP (e.g., Myo9B [74]) and other proteins (e.g., CD82 [115]) which, at least in part, are preferentially expressed in leukocytes; (ii) interact with other small GTPases and signaling pathways in a tightly regulated spatial-temporal manner [48,85], (iii) frequently regulate leukocyte-specifically expressed β2 integrins [230] to facilitate phagocytic uptake of pathogens [81], migration [130], and IS formation [150]. Future work may broaden our understanding of the regulation of the complex network of all of these factors under homeostatic conditions and in response to infection. With regard to the latter, RhoA and other small GTPases have been identified as a targets of pathogen-derived factors intended to increase pathogen uptake and to counteract (innate) immune responses [83,174]. It is tempting to speculate that the knowledge on the exact structure and mode of action of pathogen-derived factors that modulate RhoA activity may support the development of therapeutics which may counteract these microbial factors to improve immune responses. Furthermore, RhoA signaling has been identified as a target in case of autoimmune diseases [231] as well as lymphomas and leukemias caused by mutations of components of this signaling pathway [220,225].

## Abbreviations

AC: adenylate cyclase; AP-1: activator protein-1; APC: antigen presenting cell; BACURD2: BTB/POZ domain-containing adapter for Cullin3-mediated RhoA degradation 2; Bcl-6: B cell lymphoma-6; BCR: B cell receptor; BLNK: B cell linker; cAMP: Cyclic adenosine monophosphate; CCL: CC-chemokine ligand; cDC: conventional dendritic cell; CXCL: C-X-C (motif) ligand; CD: celiac disease; Cdc42: cell division control protein 42 homolog; CHS: contact hypersensitivity; CSF1: colony stimulating factor receptor 1; CYTIP: cytohesin-1 interacting protein; DC: dendritic cells; DDR1: discoidin domain receptor 1; DH: Dbl homology; EAE: experimental autoimmune encephalomyelitis ECM: extracellular matrix; EPAC: exchange protein activated by cAMP; ERM: ezrin/radixin/moesin; ERRα: estrogen-related receptor alpha; Fam65b: family with sequence similarity 65 member b; FcR: Fc receptor; FOXO1: Forkhead box O 1; G: guanine nucleotide-binding protein; GAP: GTPase-activating protein; GDI: GDP-dissociation inhibitor; GDP: guanosine diphosphate; GEF: guanosine triphosphate exchange factor; GPCR: G protein-coupled receptors; GTP: guanosine triphosphate; ICAM-1: intercellular adhesion molecule 1; IS: immunological synapse; JAK3: Janus kinase 3; KLF6: Kruppel-like transcription factor 6; LARG: Leukemia-associated Rho GEF; LIMK: Lim domain kinase; LFA-1: lymphocyte factor antigen-1; LOV: lovastatin; LPS: lysophosphaticidic acid; MAC: macrophage; mDia1: mammalian diaphanous-related formin 1; MHC: major histocompatibility complex; MIP-1α: chemokine macrophage inflammatory protein-1alpha; MLC: myosin light chain; MLCK: MLC kinase; MLCP: MLC phosphatase; MLS: myosin II regulatory light chain; MS: multiple sclerosis; Mst: mammalian sterile twenty; MT: microtubules; Myo9B: myosin 9b; Net1: neuroepithelial cell transforming gene 1; NF-κB: nuclear factor of κB; P: phosphate; PAK: p21-activated kinase; pDC: plasmacytoid dendritic cell; PH: Pleckstrin homology; PI3K: phosphatidylinositol 3-kinase; PLD: phospholipase D; PKA: protein kinase A; PKC: protein kinase C; PKN: serine/threonine-protein kinase N; PMA: Phorbol 12-myristate 13-acetate; PMN: polymononuclear neutrophils; PtdIns-4,5-P2: phosphatidylinositol-4,5-bisphosphate; PTEN: phosphatase and tensin homolog; RA: rheumatoid arthritis; Rac: Ras-related C3 botulinum toxin substrate; Rap1: Ras-related protein 1; Ras: rat sarcoma; RBD: Rho-binding domain; Rho: Ras homolog gene family; RhoA: ras homolog family member A; Rif: Rho in filopodia; ROCK: Rho-associated protein kinase; ROS: reactive oxygen species; S1P: sphingosine 1-phosphate; SLE: systemic lupus erythematosus; SWAP-70: switch-associated protein 70; Syk: protein-tyrosine kinase; TAGAP: T cell activation Rho GTPase-activating protein; TCR: T cell receptor; Tfh: follicular helper T cell; TGF-β1: transforming growth factor-beta 1; Th: T helper cell; TNF-α: tumor necrosis factor-a; Treg: regulatory T cell; WASp: Wiskott-Aldrich syndrome protein.
